
JOURNAL INFORMATION
==============================
NLM Title Abbreviation: Inter Econ
Journal ID: Inter Econ
NLM Journal ID: 101086399

Inter Economics

ISSN: 0020-5346

ARTICLE INFORMATION
==============================
PMCID: PMC8339685
PMID: 34376862
DOI: 10.1007/s10272-021-0977-6
Article ID: 977
Article version: 1
Subjects: Editorial

Structural Indicators and the Fiscal Uncertainty Principle

Breuer Christian c.breuer@zbw.eu

grid.461649.8 0000 0001 1019 0408 ZBW, Neuer Jungfernstieg 21, 20354 Hamburg, Germany
Electronic publication date: 2021 Aug 5
Print publication date: 2021
Volume: 56
Issue: 4
First page: 182
Last page: 183
Copyright: © The Author(s) 2021
License: This article is distributed under the terms of the Creative Commons Attribution 4.0 International License (https://creativecommons.org/licenses/by/4.0/).
Open Access funding provided by ZBW — Leibniz Information Centre for Economics.
License URL: https://creativecommons.org/licenses/by/4.0/

issue-copyright-statement: © The Author(s) 2021

==============================

The uncertainty principle in quantum mechanics (also known as Heisenberg's uncertainty principle) states that you cannot predict, with perfect accuracy, both the position and momentum of a particle. In economics, we realise that we can predict the values of two macroeconomic indicators, the output gap and the fiscal stance, with only limited accuracy. We cannot estimate both indicators without taking into account the other parameter's estimate. While the United States has recently used a huge fiscal stimulus to considerably increase GDP as well as potential output, Europe after the financial crisis learned how austerity not only reduced current GDP, but also potential GDP and so-called structural fiscal balances, contrary to the intention of the European fiscal rules.

One issue of simultaneous estimations of the output gap and of the "structural" fiscal indicator is endogeneity, in particular endogeneity of the output gap (or estimated potential output). While a fiscal stimulus (as currently in the US) increases GDP (and thus decreases the negative output gap), the more recent literature on the revisions of output gap estimates highlights that estimates of potential output have been reversed procyclically (Heimberger and Kapeller, 2017). Heimberger (2020) shows that one additional percentage point in predicted loss of actual output is associated with a loss in potential output of about 0.6 percentage points. Consequently, estimates of the potential output and the output gap are not independent of changes in fiscal policy.

The relationship between fiscal policy and current GDP is an established result of the modern empirical literature. Earlier studies, which estimated no or even negative fiscal multipliers and have been discussed during the euro crisis, turned out to be flawed (Guajardo et al., 2014; Breuer, 2019). Furthermore, Fatás and Summers (2018) and Gechert et al. (2019) discuss the direct relationship between fiscal policy and long-run GDP.

An increase in the fiscal deficit would increase GDP as well as potential GDP and also lead to an endogenous positive response of the primary government budget balance, depending on the effect of automatic stabilisers. The different effects will be stronger if the fiscal multiplier appears to be larger or if automatic stabilisers turn out to be higher. And the effect will be negative for negative shocks, as for example during the euro crisis or the recent COVID-19 crisis.

If the crisis worsens the economic outlook, potential output declines and structural deficits increase, thus demanding more fiscal tightening. The idea of adjusting the deficit for endogenous automatic effects of the business cycle on the government budget is the big improvement of the second generation fiscal rules, compared to the static 3% Maastricht rule. But the endogeneity of output gap estimates and thus uncertain estimates of structural balances challenge the reliability and soundness of the European fiscal framework. Currently, the rules are suspended. Researchers argue that the rules need to be revised before they are reintroduced (Breuer, 2021; Kammer and Arnold, 2021; von Weizsäcker and Krämer, 2021).

In the recent discussion of how to reform the fiscal compact and to estimate the output gap, Krahé and Sigl-Glöckner (2021) argue that potential output should be based on full employment rather than technical estimates of "structural" unemployment. In this line, Posen (2021) suggests using labour market-based cyclical indicators rather than the unobservable output gap. Already Blanchard (1990) has been sceptical about output gap estimates and suggested using unemployment as a “natural” cyclical indicator. Following this line, Fontanari et al. (2020) provide a method for how to estimate output gaps that are less prone to revisions based on the relationship between unemployment and GDP (Okun’s law).

Another idea to provide “natural” structural economic indicators would be to analyse what an indicator would look like if it maintains the status quo rather than actively shifting other indicators considerably – e.g. the long-run debt ratio – towards an arbitrary value. A “structural” deficit – e.g. of 0.5% of GDP – cannot be structural if it has considerable implications for other indicators.

Stabilising initial conditions of debt-GDP-ratios at current average levels, e.g. 100% of GDP, would allow deficits approximately as high as the nominal growth rate of GDP, well above the current limit of 0.5% of GDP of the European fiscal framework. In other words: The European fiscal framework sets deficit limits that distort debt-GDP-ratios, GDP and potential GDP away from initial conditions. Stabilising the current targets would allow a fiscal stimulus that would increase GDP as well as potential output, including an endogenous response of the fiscal balance. Assuming a lower fiscal deficit would imply that GDP, potential GDP and the output gap would be lower than expected.

A more natural structural indicator could also take into account a constant ratio of public capital to GDP, and thus require a certain amount of (net) government investment. In most European countries, this would require a higher ratio of public investment to GDP.

Further, euro area countries cannot use exchange rates to improve competitiveness and to stabilise the current account. For the current account to be balanced in the long run, a policy is required that will increase fiscal deficits or prices in surplus countries and reduce fiscal deficits in the countries running a trade deficit. Rather than pushing debt ratios to any new arbitrary level, structural indicators in a currency union should try to estimate how “structural” nominal GDP and price levels could bring real exchange rates and current account imbalances to balance. According to this view, surplus countries like Germany would need a real appreciation, taking into account the inflation target of the central bank. This would help the ECB to shift monetary policy away from the liquidity trap and the low interest environment.

On the other hand, if we ignore the systematic macroeconomic interdependencies and just fearfully try to tighten the belt of one single indicator, without any theoretical reasoning, we should not be surprised if the whole system turns out to be in disequilibrium, even when our estimated structural indicator appears to be close to balance.

Christian Breuer, ZBW — Leibniz Information Centre for Economics, Hamburg, Germany.


References

Blanchard, O. (1990), Suggestions for a new set of fiscal indicators, OECD Working Paper, No. 79.
Breuer, C. (2019), Expansionary Austerity and Reverse Causality: A Critique of the Conventional Approach, INET Working Paper, No. 98.
Breuer C Staatsverschuldung nach Corona: Rückkehr zur Goldenen Regel Wirtschaftsdienst 2021 101 1 2 3 10.1007/s10273-021-2809-5 33487763 PMC7813608
Fatás A Summers L H The permanent effects of fiscal consolidations Journal of International Economics 2018 112 238 250 10.1016/j.jinteco.2017.11.007
Fontanari C Palumbo A Salvatori C Potential Output in Theory and Practice: A Revision and Update of Okun’s Original Method Structural Change and Economic Dynamics 2020 54 247 266 10.1016/j.strueco.2020.04.008
Gechert S Horn G Paetz C Long-Term Effects of Fiscal Stimulus and Austerity in Europe Oxford Bulletin of Economics and Statistics 2019 81 3 647 666 10.1111/obes.12287
Guajardo J Leigh D Pescatori A Expansionary Austerity? International Evidence Journal of the European Economic Association 2014 12 4 949 968 10.1111/jeea.12083
Heimberger P Potential Output, EU Fiscal Surveillance and the COVID-19 Shock Intereconomics 2020 55 3 167 174 10.1007/s10272-020-0895-z 32536715 PMC7276112
Heimberger P Kappeller J The performativity of potential output: pro-cyclicality and path dependency in coordinating European fiscal policies Review of International Political Economy 2017 24 5 904 928 10.1080/09692290.2017.1363797
Kammer A Arnold N Europe’s COVID-19 Crisis Response: A Race Well Run, But Not Yet Won? Inter-economics 2021 56 4 194 196 10.1007/s10272-021-0981-x PMC8339699 34376865
Krahé M Sigl-Glöckner P Die Definition einer zukunftsfähigen Finanzpolitik Wirtschaftsdienst 2021 101 7 497 500 10.1007/s10273-021-2954-x
Posen A Fiscal Success During COVID-19 Says Believe the Good News Intereconomics 2021 56 4 190 193 10.1007/s10272-021-0980-y PMC8339686 34376864
von Weizsäcker, C.-C. and H. M. Krämer (2021), Saving and Investment in the Twenty-First Century, Springer.
